# Effect of Different Ceramic Materials on Fatigue Resistance and Stress Distribution in Upper Canines with Palatal Veneers

**DOI:** 10.1055/s-0041-1740225

**Published:** 2022-02-03

**Authors:** Isabela Vitelli Tanaka, Joao Paulo Mendes Tribst, Lais Regiane Silva-Concilio, Marco Antonio Bottino

**Affiliations:** 1 Department of Dental Materials and Prosthodontics, Institute of Science and Technology, São Paulo State University, São José dos Campos, São Paulo, Brazil; 2 Department of Dentistry, University of Taubaté, Taubaté, São Paulo, Brazil

**Keywords:** dental materials, dental veneers, fatigue, finite element analysis

## Abstract

**Objective** The aim of this study was to evaluate, by means of a fatigue life test, different ceramic materials used in palatal veneers to restore the canine guidance.

**Materials and Methods** Forty-five standardized anatomical preparations were made in extracted healthy human canines with 1.2 uniform thickness. Samples were scanned, restorations were designed and milled in polymer-infiltrated ceramic network (PICN, Vita Enamic), zirconia-reinforced lithium silicate (ZLS, Vita Suprinity), and high translucent yttrium oxide-stabilized tetragonal zirconia (YZHT, Vita YZHT). Dental preparations were etched, restorations were processed according to the manufacturers' recommendations, and adhesively cemented. Then, three samples of each group were tested with load-to-fracture to determine the fatigue parameters. In addition, the palatal veneers stresses were evaluated using numerical models through finite element analysis.

**Results** The mean of the monotonic test for PICN, ZLS, and YZHT was 674.18 N, 560.5 N, and 918.98 N, respectively. The StepWise test was performed until specimen fracture or until suspension of the test after 1.2 × 10 ^6^ cycles. Regarding survival, using the Kaplan–Meier method, PICN presented results for the mean and median of 245.21 N and 225 N, respectively; ZLS had an average of 175.76 N and a median of 168 N, and YZHT with an average of 383.30 N and a median of 366 N. Regarding the Weibull method, PICN showed results of 5.43 β and 264 η for form and scale, respectively; ZLS had 36.14 β for form and 380.67 η for scale; and YZHT presented 4.95 β for form and 417.38 η for scale. The highest stress value was calculated for YZHT, ZLS, and PICN, respectively.

**Conclusions** It was possible to conclude that all tested materials have the possibility of being used for rehabilitation of upper canines' palatal surface.

## Introduction

The stomatognathic system presents three functional units with actions coordinated by the central nervous system, namely, the temporomandibular joint, masticatory muscles, and dental occlusion, which involve the skeletal components (maxilla and mandible) and the dental arches. This set of actions promotes basic and functional activities such as swallowing, chewing, sucking, and phonation. 1

For these structures to work in harmony, it is necessary to have the natural dentition and disocclusion guides, which can occur by the canine guidance or in-group function. In canine guidance, the contact during lateral movement occurs only between upper and lower canines, while in group guidance, the movement can be observed in the posterior region with at least two premolars on the working side. 2

The disocclusion guide in the canine is the ideal contact in laterality on the working side since the performed movement acts as a force breaker reducing the activity of the jaw elevator muscles, preventing pain and parafunctions. 3 This event is explained by the location of the canine in the arch, its voluminous roots, bone reinforcement, palatal concavity, and steep cusp. 3 In addition, other important functions such as speech, social interaction, chewing, and swallowing can be affected by the occlusion and muscle activity function, thereby protecting the stomatognathic system from dysfunction and damage. 4

Currently, with the preservation of dental elements and population aging, the extremely worn dentition is considered a major clinical and aesthetic issue. 5 Dental wear facets can be found in the population, varying from small sites located only in enamel to large destruction also affecting dentin. These facets can also vary in terms of location, found in a single tooth or generalized in several elements. 5 They can occur by three mechanisms: attrition that is characterized as the wear of the hard tooth structures through tooth contact, abrasion that occurs through the action of abrasive agents between tooth surfaces, and erosion that means the loss of tooth structure against acidic agents. 5 6 Gradual wear of occlusal and incisal surfaces is considered physiological as long as it occurs slowly. 7

Thus, the principle of minimally invasive dentistry seeks to return function and aesthetics to the patient with the least possible wear to the tooth structure through conservative techniques. This concept is possible by the new range of restorative materials and cementing agents. Clinical success for cases performed with this philosophy occurs through correct indication and planning, otherwise biological damage such as gingival inflammation over the contour of the prosthetic piece can be observed. 8

The loss of protection guides deserves attention from the dental surgeon, especially when the patient has parafunctional habits. Thus, the construction of a lingual veneer for correction or creation of disocclusion guides and aesthetic recovery of lost prosthetic crowns can be indicated. 9

One of the ways to recover the lost tooth structure is through the use of biomaterials capable of mimicking the characteristics of the dental tissues. In this sense, the worn palate surface can be recovered using ceramic or resin materials; the choice of material is up to the dental surgeon's assessment and planning. 10 However, how different materials can modify the stress magnitude and restoration reliability during the loading of upper canines with palatal veneers has not been reported in the literature yet.

As a way to rehabilitate the patient who lost the protection guides due to bruxism, some dental ceramics are available on the market that are indicated by combining aesthetics and resistance in the same material: Zirconia-reinforced lithium silicate glass ceramic (ZLS, Vita Suprinity [ZLS]; Vita Zahnfabrick, Bad Säckingen, Germany) has a reliable Weibull distribution for clinical use, and an intermediate elastic modulus of 70 GPa. 11 12

The high translucency zirconia (Vita YZHT; Vita Zahnfabrick, Bad Säckingen, Germany), which was launched with the aim of combining the good mechanical microstructural properties of zirconia with optical improvements, has an elastic modulus of 210 GPa and can be a restorative alternative to promote strong restorations. 13

The polymer-infiltrated ceramic network (PICN, Vita Enamic; Vita Zahnfabrick, Bad Säckingen, Germany) exhibits a mechanical behavior similar to dentin, with an elastic modulus of 30 GPa. PICN also has the advantage of having superior resistance compared with tooth enamel, since the progression of cracks in this material is prevented by the interface between polymer and ceramic networks. 14 15

Thus, the aim of this study was to evaluate the fatigue strength of human canines restored with palatal veneers made with different ceramic materials.

## Materials and Methods

### Sample Preparation

This study was submitted and approved by the Ethics and Research Committee of the Science and Technology Institute of UNESP, São José dos Campos, São Paulo, Brazil.

The human canines used in this study were donated by the Human Teeth Bank of Universidade Paulista (UNIP, São Paulo, Brazil) and were selected with the criterion of being intact and without any type of restoration. Only adult canines from patients between 18 and 60 years old were used. All teeth came clean and stored in an aqueous medium below 5°C.

**Fig. 1:**
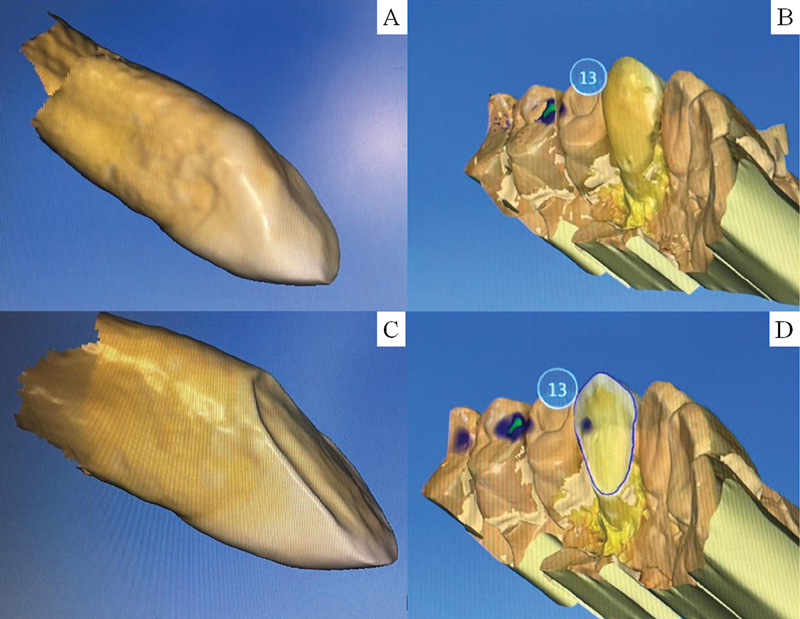
Virtual impression (computer-aided design) from a representative sample before ( **A** and **B** ) and after preparation ( **C** and **D** ) and delimitation of the restoration margin.

At first, all teeth were scanned with an intraoral scanner (CS3600, Carestream, Atlanta, Georgia, United States ( Fig. 1 ) to obtain each anatomy prior to the preparation. Then, 45 standardized anatomical preparations were made by a single operator, in extracted healthy human canines with 1.2 mm-thick uniform wear, using counter multiplier angle 1: 4.5 with refrigeration (Alegra Led G, Wilcos, Petrópolis, Rio de Janeiro, Brazil) and diamond burs with spherical and candle flame shape (KG Sorensen, Cotia, São Paulo, Brazil). To standardize the preparation, total shape of a 1.2 diameter diamond bur was used and only one experienced operator performed all the preparations. This preparation measurements were chosen because it is a value in the range of the three materials' indication. According to the literature, the average enamel thickness in canine tooth is of 827 ± 196 μm. 16 Therefore, according to a uniform value of enamel thickness in the palatal face, the present preparation allowed ∼0.4 μm of dentin wear. However, the restoration margin remained in enamel regardless the material group. Each sample received a preparation design with chamfer finish line.

During the execution of the procedures, all teeth were randomly separated, with no prior selection for each group.

After the preparations were made, all teeth were again scanned with the intraoral scanner (CS3600, Carestream, Atlanta, Georgia, United States). The standard triangle language file was sent to the CAD (computer-aided design) software (Dental CAD - Exocad GmbH, Darmstadt, Germany) where the delineation of the preparation margin and insertion axis of the restoration were performed. The restorations were designed matching the images before and after preparation (Biocopy). All restorations showed an internal relief of 70 µm. Then, the virtual structures were sent to the milling unit (Cerec MC XL, Dentsply Sirona, Erlangen, Germany) to be milled in different restorative materials. The preparations and respective CAD models were randomly divided into three experimental groups, according to the ceramic material.

Veneers were manufactured in polymer-infiltrated glass ceramic, PICN (VITA Enamic, Vita Zahnfabrik, Germany), ZLS (Vita Suprinity, Vita Zahnfabrik, Germany), and high translucent yttrium oxide-stabilized zirconium, YZHT (Vita YZHT, Vita Zahnfabrik, Germany). For milling, two cylindrical tips were used, one of 1.6 mm in diameter that was mounted on the left arm of the milling machine for milling the external surface of the structures and another of 1.2 mm in diameter was mounted on the right arm for the internal surface, which were both replaced after 20 milled blocks.

After milling, all restorations were finished, processed, and polished with the aid of respective ceramic systems; then they were cleaned in an ultrasonic bath with isopropyl alcohol for 10 minutes. PICN restorations do not require crystallization/sintering because their manufacturing process already contains the final ceramic phase, which facilitates the milling process.

ZLS restorations were crystallized at ceramic furnace (Programat EP5000, Ivoclar vivadent, Schaan, Liechtenstein). YZHT structures were milled with ∼20% increase in final dimensions to compensate the shrinkage after sintering, then were sintered in high temperature furnace (Sirona in Fire HTC, Dentsply Sirona, Bensheim, Germany) according to the manufacturer's recommendations ( Table 1 ).

**Table 1:** YZHT sintering parameters and ZLS crystallization parameters

Furnace parameters	ZLS	YZHT
Initial temperature (°C)	400	25
Closing time (minutes)	8	1
Heating rate (°C/minutes)	55	17
Final temperature (°C)	840	1450
Maintenance time T final (minutes)	8	120
Oven opening temperature (°C)	680	200

Abbreviations: ZLS, zirconia-reinforced lithium silicate; YZHT, yttrium oxide-stabilized tetragonal zirconia.

As a surface finishing method, all restorations were polished with fine-grained and extra-fine-grained diamond rubbers (Exa-cerapol; Edenta, São Paulo, Brazil) recommended by the manufacturer of the respective ceramic systems. 17

All samples were embedded in 25 mm PVC (polyvinyl chloride) tubes containing acrylic resin (JET, Classic Dental Articles, Campo Limpo Paulista, São Paulo, Brazil) 2 mm below the cervical end of the preparation. The PVC base was angled at 30 degrees to keep the specimens at the ideal angle during the mechanical test. 18 The oblique incidence of loading aims at reproducing a critical stress distribution during the sample testing.

### Cementation Procedure

Dental preparations were etched with 37% phosphoric acid for 30 seconds (Condac, FGM, Joinville, Santa Catarina, Brazil) and washed with water and dried with oil-free air jets. Self-etching primer (Tooth Primer Panavia V5, Kuraray Noritake Dental, Tokyo, Japan) was applied with a microbrush for 20 seconds and dried with a light jet of air.

For the surface treatment of ceramics, etching was performed with 5% hydrofluoric acid for 20 seconds in ZLS (Condac, FGM, Joinville, Santa Catarina, Brazil) (Itthipongsatorn, Srisawasdi, 2020) and for 60 seconds in PICN, 15 then the restorations were washed with a water/air jet for 20 seconds and dried with an air jet for 30 second. As YZHT restorations are acid resistant, 19 they were blasted with 50 micron aluminum oxide particles (Bio-Art, São Carlos) for 20 seconds (2.8 bar, 10 mm distance), washed in ultrasound with isopropyl alcohol for 10 minutes and dried.

**Fig. 2:**
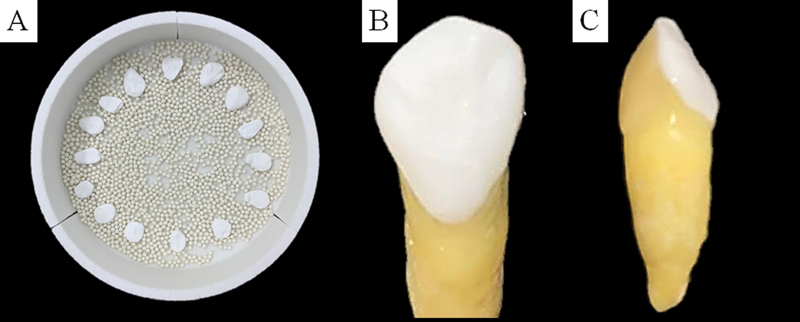
Yttrium oxide-stabilized tetragonal zirconia samples prior to the sintering process ( **A** ), and after cementation in palatal view ( **B** ) and lateral view ( **C** ).

Subsequently, all restorations received silane application (Clearfil Ceramic Primer Plus, Kuraray Noritake Dental, Tokyo, Japan) according to the manufacturer's recommendations. Cementation was performed with resin cement (Panavia V5 Kuraray Noritake Dental, Tokyo, Japan). The cement was applied to the intaglio surface of the restoration, and taken into position under a load of 750 g. The set was light cured (Valo, Ultradent, South Jordan, Utah, United States) with a light intensity of 1,400 mW/cm ^2^ for 40 seconds ( Fig. 2 ). The restorations were stored in an oven at 37°C and after 24 hours, they were submitted to the fatigue test.

### Compressive Test

**Fig. 3:**
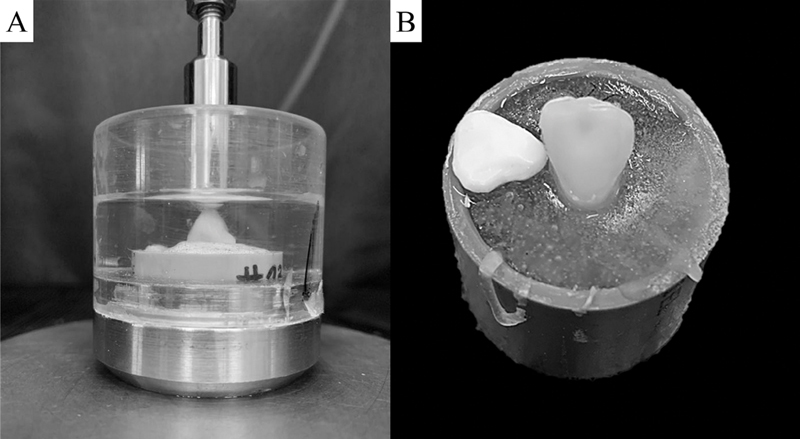
Universal testing machine during the compressive test ( **A** ). Yttrium oxide-stabilized tetragonal zirconia representative sample right after monotonic test with adhesive failure ( **B** ).

Initially, three restorations from each group were submitted to the load-to-fracture test, under compressive load with samples immersed in distilled water ( Fig. 3 ). For this, the specimens were positioned in a universal testing machine (EMIC DL 1000; EMIC, São José dos Pinhais, São Paulo, Brazil). Then, an increasing load using a 1000 kgf load cell and a speed of 1.0 mm/min was applied. The load application was performed with a 4.6 mm diameter steatite piston in the palatal surface of the restorations. The choice of this material as antagonist was due to its elastic modulus close to human dental enamel. 20 The mean of the maximum load values, in N, for each experimental group was used as the basis for determining the parameters for the fatigue test ( Table 2 ). 15 19 21

**Table 2:** Load-to-fracture average values (N) calculated to determine the fatigue profile

Material	Value for each sample	Average	Standard deviation
PICN	716.37 N 633.96 N 672.21 N	674.18N	51.65
ZLS	625.26 N 546.32N 510.16N	560.58N	13.32
YZHT	972.32N 943.41N 841.22N	918.98N	88.53

Abbreviations: PICN, polymer-infiltrated ceramic network; ZLS, zirconia-reinforced lithium silicate; YZHT, yttrium oxide-stabilized tetragonal zirconia.

### StepWise Fatigue Test

An adaptation of the StepWise test was used, similar to previously reported studies. 2 3 4 5 6 7 8 9 10 11 12 13 14 15 16 17 18 19 20 21 22 23 All specimens were submitted to fatigue cycling protocol, which consists of the application of 5,000 cycles at a low load to accommodate the applicator to the specimen surface, followed by progressive load cycles, in steps, until the specimens fracture or the suspension of the specimen test after 1.2 × 10 ^6^ cycles.

For the present study, the load applicator consisted of a steatite piston fixed to the fatigue equipment to simulate an antagonistic tooth, since steatite has an elastic modulus of 120 GPa, considered similar to that of human dental enamel. 20

Twelve specimens from each experimental group with horizontal load displacement of 2 mm were tested. Loading was performed with a frequency of 2 Hz in a fatigue tester (Biocycle V2 equipment, Biopdi, São Carlos, São Paulo, Brazil). The progressive steps were defined based on the results of the monotonic test for each material.

**Fig. 4:**
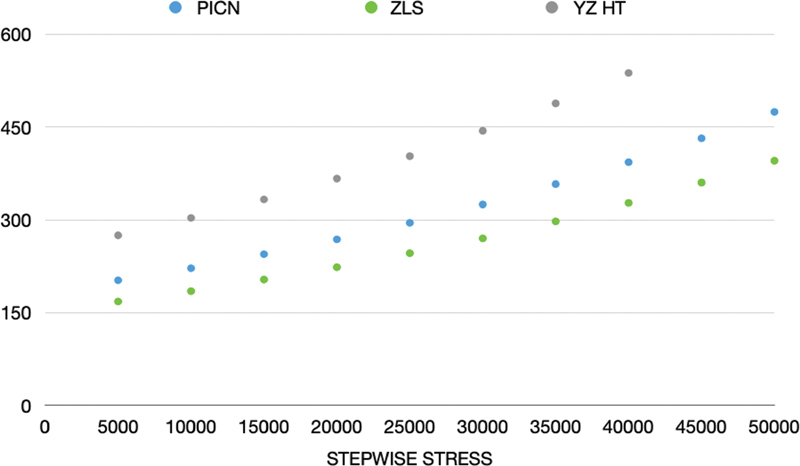
Graph with fatigue profiles referring to the accelerated life test of the polymer-infiltrated ceramic network (PICN), zirconia-reinforced lithium silicate (ZLS), and Yttrium oxide-stabilized tetragonal zirconia (YZHT) groups.

The graphs below illustrate the progressive load steps with an increase of 10% from the initial value of the mean value of the monotonic test in N ( Fig. 4 ).

The presence of cracks and/or fractures was checked with the aid of adequate lighting every 2.5 × 10 ^5^ cycles, and after 5 × 10 ^5^ cycles the test was interrupted for evaluation under a stereomicroscope.

### Fracture Analysis

The samples were analyzed under a stereomicroscope (Discovery V20 -Zeiss, Jena, Germany) to assess cracks and fractures with magnification of 10x, 25x, and 45x. Failures were classified according to type: (1) crack or partial fracture of the restoration, (2) catastrophic fracture of the restoration in multiple fragments, and (3) detachment of the facet.

### Data Analysis

**Fig. 5:**
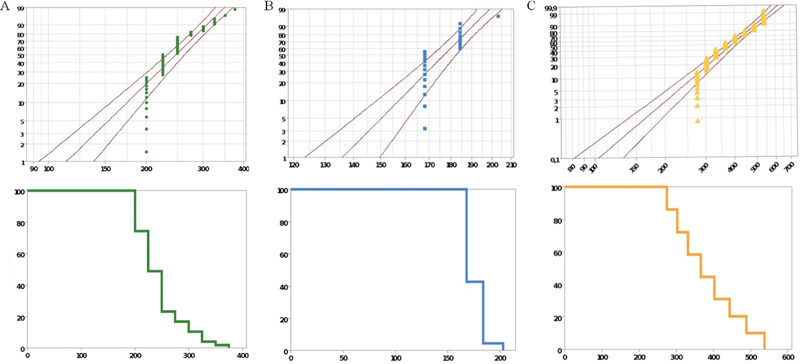
Survival probability graph with Weibull and Survival load using Kaplan–Meier method; for polymer-infiltrated ceramic network ( **A** ), zirconia-reinforced lithium silicate ( **B** ), and yttrium oxide-stabilized tetragonal zirconia ( **C** ).

The number of cycles to failure was used for survival analysis by Kaplan–Meier tests without direct comparison between materials because of the use of different fatigue profiles. The probability of failure was calculated for different step intervals (Minitab, v16.1.0; Minitab, LLC). The two-parameter Weibull failure probability analysis ( Table 3 ) provided the β value (β), which is a shape parameter and describes the behavior of the failure rate over time. The parameter eta (η) represents the characteristic life of the samples, in which 63.2% of failures occur ( Fig. 5 ).

**Table 3:** Representing shape and scale values following the Weibull modulus

Material	Shape	Scale
PICN	5.43	264
ZLS	36.14	380.67
YZHT	4.95	417.38

Abbreviations: PICN, polymer-infiltrated ceramic network; ZLS, zirconia-reinforced lithium silicate; YZHT, yttrium oxide-stabilized tetragonal zirconia.

### Finite Element Analysis

The analysis of stress distribution in palatal restorations was performed using the two-dimensional finite element method, comparing the different types of ceramics. Two-dimensional models were obtained through lateral plan image of a representative standard tooth. 24 25

The files in bitmap image format were transferred to the CAD software (Rhinoceros 5.0, McNeel North America, Seattle, Washington, USA), where they were used as background references for designing the models, following the BioCAD protocol. 26 At this stage, the models were generated from the lines drawn on the image following the visible anatomical references. The models were exported to the computer aided engineering software (ANSYS, 17.0, Ansys, Canonsburg, Pennsylvania, United States) for the preprocessing of the finite element analysis, and converted into two-dimensional plane models for the plane stress analysis.

Then, the mechanical properties of the materials were assigned ( Table 4 ). The interfaces were considered perfectly bonded for all groups and the cement thickness of 70 μm was considered similar to the *in vitro* setup. All materials were considered isotropic, linear, and homogeneous for the mechanical static structural analysis.

**Table 4:** Mechanical properties (elastic modulus and Poisson ratio) of the materials simulated in this study

Simulated material	Elastic modulus (GPa)	Poisson's coefficient
Enamel	84.1	0.33
Dentin	18.6	0.32
Resin cement	6	0.3
ZLS	70	0.23
YZHT	200	0.31
PICN	34.7	0.28
Periodontal ligament simulator	0.15 (×10 ^−3^ )	0.3
Support resin	2.7	0.35

Abbreviations: PICN, polymer-infiltrated ceramic network; ZLS, zirconia-reinforced lithium silicate; YZHT, yttrium oxide-stabilized tetragonal zirconia.

As boundary conditions, the models were fixed at the base of the fixation cylinder, and the load was applied to the palatal face, in the cingulum region with a force of 100 N, 30 degrees. 27 The load was applied above the cingulum, between the lingual lobe and lingual ridge in the most convex region. The chosen analysis criterion was the evaluation of the maximum main stress (MPa), in which the tensile values are shown in warm colors in the generated colorimetric stress maps.

## Results

The Kaplan–Meier method (log-rank *p* < 0.001) was applied with a confidence level of 95%. Table 3 shows, through the Weibull modulus, the values of the shape and scale parameters for each material group. In the PICN group, shape was 5.43 and scale was 264, ZLS had a shape of 36.14 and a scale of 380.67, and YZHT had a shape of 4.95 and 417.38. Higher values for shape represent failure rate that increases with time, which would be indicative of future problems regarding wear/failure or even end of material life. However, higher values for scale represent the survival reliability defining the data distribution during the loading.

In the survival graphs through the Kaplan–Meier method, values close to the mean and median for the samples of the three groups (PICN, ZLS, and YZHT) were 245.21 and 225; 175.76 and 168, and 383.3 and 366, respectively.

**Fig. 6:**
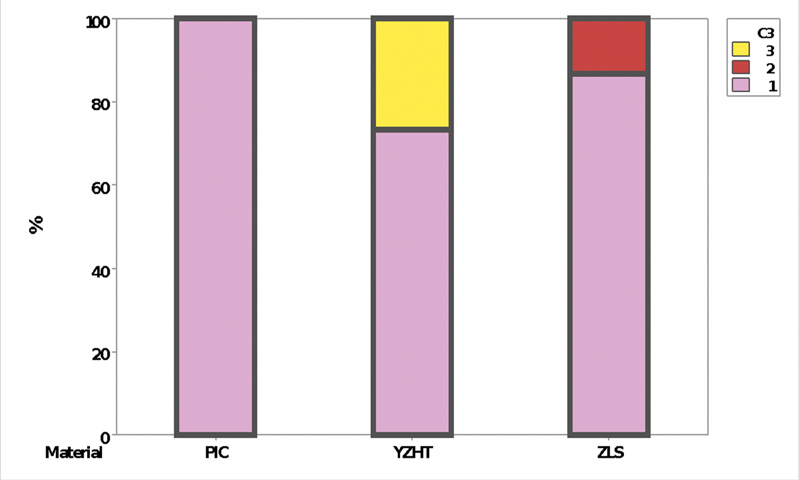
Failure mode represented by numbers: 1—crack or partial fracture of the restoration, 2—catastrophic fracture of the restoration in multiple fragments, and 3—detachment of the veneer. PICN, polymer-infiltrated ceramic network; ZLS, zirconia-reinforced lithium silicate; YZHT, yttrium oxide-stabilized tetragonal zirconia.

All three groups had a crack or partial fracture of the restoration; only the ZLS group presented catastrophic failure in multiple fragments; and the YZHT group, in addition to the crack or partial fracture of the restoration, presented facet debonding. ( Fig. 6 )

**Fig. 7:**
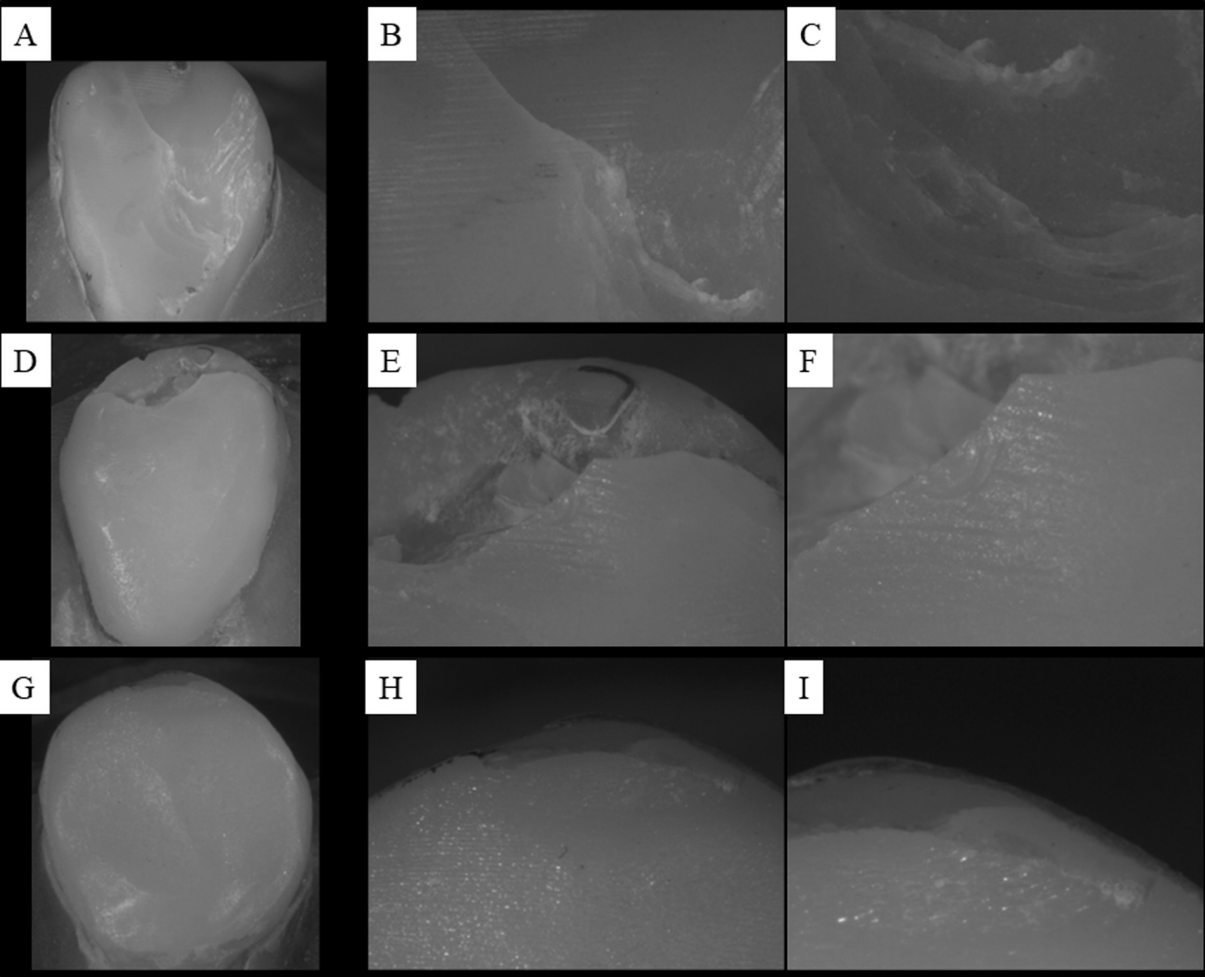
Sample after fatigue test. It is possible to observe cracks in the restoration in the analysis under a stereomicroscope with respective magnifications of 10x/25x/45x. In sequence polymer-infiltrated ceramic network ( **A** – **C** ), ZLS ( **D** – **F** ), and yttrium oxide-stabilized tetragonal zirconia ( **G** – **I** ).

Assuming the maximum principal stress criterion, which demonstrates the resulting tensile stress on the structures involved, it is possible to separately observe tooth, restoration, and resin cement in Fig. 7 (A–I) respectively. In Fig. 7 (D–F) , it is possible to observe a similar trend between the models, with no visible difference for the qualitative results of stress.

However, for the result of stress in the palatal restoration, there is a visible difference in the magnitude of maximum stress magnitude in the intaglio surface of the veneer. Basically, the tensile stress accumulated in the adhesive interface region is proportional to the elastic modulus of the restorative material.

**Fig. 8:**
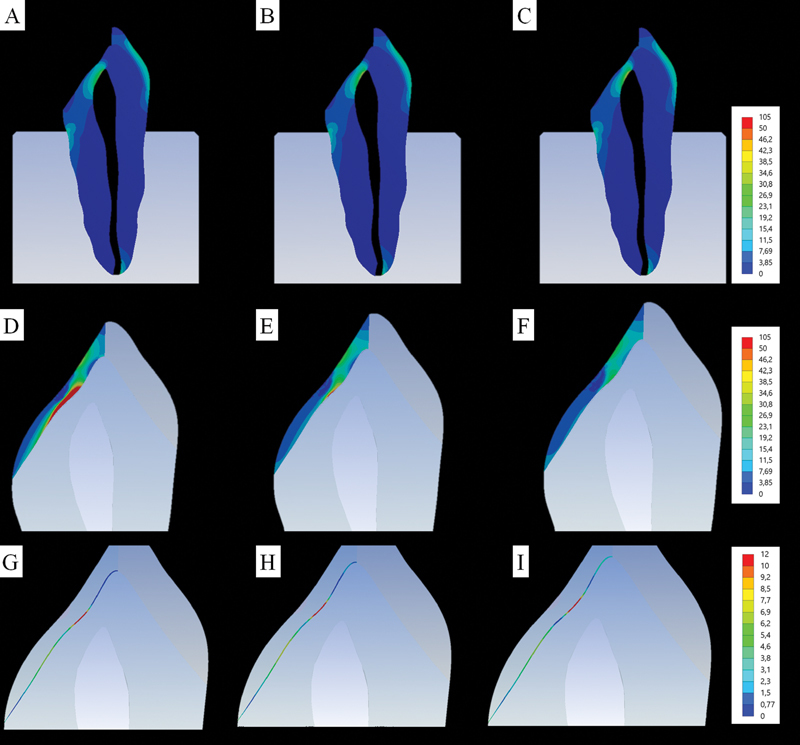
Maximum principal stress (MPa) in dental substrate, at the veneer and the cement layer for yttrium oxide-stabilized tetragonal zirconia veneer ( **A** ), zirconia-reinforced lithium silicate veneer ( **B** ), and polymer-infiltrated ceramic network veneer ( **C** ).

As for the resin cement layer, small differences are noticed in relation to the highest values in the red fringe of the numerical scale. However, in the apical region of the preparation, a small but visible difference can be observed between YZHT and PICN, with greater magnitude for PICN ( Fig. 8 ).

## Discussion

In this study, the stress concentration and fatigue survival of different ceramic materials for the replace of the canine guidance through palatal veneers have been evaluated. The hypothesis that materials have different Weibull modulus as well as different stress magnitudes was proven.

Despite the wide variety of materials available that can be used for this type of treatment, when indicating a material such rehabilitation, it is necessary to carefully assess the patient's current oral condition, such as parafunctional habits, amount of remnant, and whether there will be involvement of both arches in the rehabilitation.

There are several reasons that can lead to wear on the palatal surface of human dentition, and the three main factors are behavioral (eating habits, acidic drinks, gastric problems, drugs), biological (salivary pH, dental structure, soft tissue versus hard tissue), and socioeconomic (general health, education, knowledge, habits in general). 28 Basically, the wear is physiological and it will significantly increase with age; for example, the prevalence of dental erosion in Israel has increased from 36.6% among ages 15 to 18 to 61.9% between 35 and 49 years and 100% between 50 and 74 years. 29 In addition, the frequent exposure of dental structures to acids leads to a permanent and visible loss of dental tissues. 29

Regardless of the etiological factor capable of modifying the morphology of the tooth structure through unwanted wear, the present study demonstrates that prosthetic rehabilitation with a ceramic veneer is a viable alternative with acceptable characteristic strength, regardless of the evaluated material.

Generally, in cases of pathological wear, the proposed treatment can be through a more conservative technique, according to the literature, 30 by increasing the vertical dimension of the patient's occlusion, recovering interocclusal space and maybe not requiring extensive dental preparations. 30 This philosophy of “add and not remove” not only requires more frequent maintenance but also preserves more of the original structure of the tooth, which is already naturally worn and allows less associated dental treatments, such as an endodontic treatment.

Furthermore, previous authors 31 proved the success of oral rehabilitation in a young patient, due to dental erosion, in an extremely conservative way, thus achieving minimal dental preparation and preservation of tooth vitality. It is important to notice that the idea of preservation of vital structures, especially in a young patient, shows better prognosis for the teeth in the long-term.

Therefore, during the preparation of the samples in the present study, a condition in which the replacement of the lost structure through ceramic biomaterials was assumed, whether it would be sufficient as a therapeutic option. However, it is noteworthy that under different conditions from the clinical parameters simulated here, new results may be observed, affecting the prognosis of the case.

For Muts et al, 32 it is difficult to choose the best technique or material when it comes to oral rehabilitation due to the complexity of the treatment. Thus, it is suggested that for the planning of an oral rehabilitation, a diagnostic wax-up should be performed, in relation to the central occlusion position, and if possible, an increase in the occlusion dimension.

In the present study, the different materials evaluated presented a minimum thickness indicated by the manufacturer, aiming at guaranteeing the dental morphological recovery along with the structure. Thus, a more conservative treatment was evaluated, with the restoration limited to the worn region to recover the shape and function without involving the other sides of the tooth. This condition was previously present in previous studies. 30 31 However, the evaluation of different materials had not been performed before. When comparing the three types of ceramics used, we must keep in mind the differences between them. PICN is characterized by being a polymer-reinforced glass ceramic, which has advantages in terms of resistance to chewing force without causing wear on the antagonist 33 and by its outstanding performance during cyclic fatigue due to its polymeric network. 15 34 In the present study, this material showed higher survival values when compared with the ZLS samples but it did not resist as much compared with the YZHT results.

YZHT ceramic is an evolution of the zirconia family as it presents greater translucency when compared with first and second zirconia generation, and it also has better mechanical properties. 34 The high flexural strength is one of the great advantages of the material, justifying why this material did not present catastrophic fractures in the present study. The veneer debonding can be a promising failure type presenting the advantage of bonding the same veneer. In situations of detachment, it is possible to carry out the cementation of a zirconia structure, without necessarily manufacturing a new veneer, in cases where the part is between 1 and 2 mm thick. 35

In the present study, during simulated chewing, 75% of the YZHT samples suffered debonding, corroborating previous literature reports. In addition to the lower bond strength of polycrystalline ceramics compared with the other evaluated materials, 36 these failures can also be explained by the higher stress magnitude calculated in the adhesive interface region observed by finite element analysis. This finding corroborates a previous report, which observed similar biomechanical behavior for buccal veeners. 37

ZLS is a glass material that combines aesthetic and mechanical characteristics, but compared with the other materials, it presented a superior Weibull modulus, in terms of shape, suggesting a possible failure regarding wear and consequently, end of life. That is, over time failures will increase. These findings are in agreement with previous study, 19 which also evaluated the same materials simulated in this study and concluded that the higher the elastic modulus of the ceramic (PICN < ZLS < YZHT), the greater the stress concentration in the restoration.

In addition, the literature demonstrates that ZLS is a sensitive material in terms of surface characteristics, and may present early failures when not properly polished. 12 Continuing in this sense, during the incidence of cyclic loads, the group restored with ZLS presented a predominance of catastrophic failures, thus evidencing the fragility of this brittle material in comparison to the others; however, with characteristic resistance values higher than the maximum human bite load with a value of 127 kg. 38 However, patients with chronic bruxism have much more developed masseter muscles and will produce much higher forces than 127 kg. The maximum anterior bite forces can reach up to 370 N in protrusion and edge-to-edge position. 39 40 Therefore, the parafunctional habits must be resolved before any restoration is placed. In addition, decayed tissue removal, periodontal treatments, recovery of the vertical dimension of occlusion with provisional restorations, and other urgent and immediate dental treatments must be done before placement of ceramic restorations in cases with severe wear due to pathological oral habits.

During the cyclic fatigue used in this study, StepWise test, all samples were positioned so that the steatite piston could slide in the palate-incisal direction, immersed in distilled water and were subjected to an initial load corresponding to 30% of the monotonic value. The increase in load was progressive by 10%, as there were no signs of fracture, and the test was suspended due to cracks, fractures, and/or debonding of the veneer. Steatite was previously reported in the literature as a substitute for dental enamel during *in vitro* tests allowing the proper standardization of the antagonist. 20 39 40 Nevertheless, these conditions are limited to simulated parameters and do not present the same conditions founds in the oral environment.

According to the literature, the StepWise test is an accelerated fatigue method used to test brittle ceramic materials, to assess fatigue behavior and the damage accumulation over time, being an important method to detect failures in ceramic biomaterials. 19 41 In addition, the fractures produced in the StepWise test were similar to those observed *in vivo* and with excellent indication for the study of fatigue in ceramic restorations. 21

Regarding the failure mode of the materials, it is noted that three types of failures were predominant: crack/fracture, catastrophic, and veneer debonding. It is known that ceramic materials fail due to the slow growth of cracks that promote critical fractures. 42 This event can be explained by the theory of the ''weakest link'', where the fracture always propagates from the largest origin with highest stress magnitude, with the distribution of size, shape, and origin differing for each material and distributed according to the defect size distribution. 42 As for Melo et al, 43 another factor that also affects the slow growth of cracks is the salivary pH variation, which reflects independently for each ceramic material, occurring in clinical situations. As no alterations in pH were performed in the present study, it is possible to assume this is not a reason for the observed failure mode.

The finite element analysis of this study was extremely important to complement the statistical results, and thus it shows more clearly the stress in the adhesive interface region. Previous studies demonstrated that the stress concentration region corresponds to the region of failure origin proportional to the material's modulus of elasticity. 19 37 44

Previous finite element analysis simulations have already demonstrated the influence of restorative material for anterior and occlusal veneers; however, the present study sought to study palatal veneers, aiming at this new approach to oral rehabilitation with the lack of scientific literature about it.

Finite element studies have concluded that the combination of ceramic and cement can affect the performance of ceramic veneers in upper central incisors. 37 45 46 In addition, Tribst et al 45 evaluated occlusal veneers in posterior teeth and concluded that than the higher elastic modulus of the material than higher the stress magnitude. Corroborating with that, in the present study the PICN samples showed lower stress concentration in relation to the restoration, but not in relation to the cement layer.

As for the limitations of this study, we can mention the use of only intact natural teeth, without considering teeth with previous restorative procedures on it. In addition, the fatigue test was performed in the absence of pH and temperature variations. The restoration groups comparison could not be performed due to the different fatigue profiles calculated after the load-to-fracture. The biofilm simulations should also be considered limitations of this study as well as other materials on the antagonist tooth during the loading incidence. The stress map was resultant from a linear analysis with isotropic materials and perfected bonded interfaces. Further studies should be performed to elucidate the biomechanical behavior from different fatigue parameters and clinical conditions.

## Conclusion

With the limitations of this *in vitro* study, based on the individual fatigue parameters of each material evaluated, all the materials tested have the possibility of being used in the rehabilitation of the palatal surface of maxillary canines. However, YZHT and PICN showed promising results of characteristic strength and reliability, being, therefore, the suggested materials for this type of rehabilitation.

## References

[1] Cuccia A Caradonna C The relationship between the stomatognathic system and body posture Clinics (São Paulo) 2009 64 01 61 66 19142553 10.1590/S1807-59322009000100011 PMC2671973

[2] Akören A C Karaağaçlioğlu L Comparison of the electromyographic activity of individuals with canine guidance and group function occlusion J Oral Rehabil 1995 22 01 73 77 7897562 10.1111/j.1365-2842.1995.tb00213.x

[3] D'Amico A Functional occlusion of the natural teeth of man J Prosthet Dent 1961 11 05 899 915

[4] Kulmer S Ruzicka B Niederwanger A Moschen I Incline and length of guiding elements in untreated naturally grown dentition J Oral Rehabil 1999 26 08 650 660 10447819 10.1046/j.1365-2842.1999.00430.x

[5] Malkoc M A Sevimay M Yaprak E The use of zirconium and feldspathic porcelain in the management of the severely worn dentition: a case report Eur J Dent 2009 3 01 75 80 19262736 PMC2647964

[6] Grippo J O Simring M Schreiner S Attrition, abrasion, corrosion and abfraction revisited: a new perspective on tooth surface lesions J Am Dent Assoc 2004 135 08 1109 1118, quiz 1163–1165 15387049 10.14219/jada.archive.2004.0369

[7] Bourdiol P Hennequin M Peyron M A Woda A Masticatory adaptation to occlusal changes Front Physiol 2020 11 263 32317982 10.3389/fphys.2020.00263 PMC7147355

[8] Farias-Neto A Gomes E M Sánchez-Ayala A Sánchez-Ayala A Vilanova L S Esthetic rehabilitation of the smile with no-prep porcelain laminates and partial veneers Case Rep Dent 2015 2015 452765 26568893 10.1155/2015/452765 PMC4628695

[9] Miralles R Canine-guide occlusion and group function occlusion are equally acceptable when restoring the dentition J Evid Based Dent Pract 2016 16 01 41 43 27132554 10.1016/j.jebdp.2016.01.029

[10] Magne P Schlichting L H Maia H P Baratieri L N In vitro fatigue resistance of CAD/CAM composite resin and ceramic posterior occlusal veneers J Prosthet Dent 2010 104 03 149 157 20813228 10.1016/S0022-3913(10)60111-4

[11] Elsaka S E Elnaghy A M Mechanical properties of zirconia reinforced lithium silicate glass-ceramic Dent Mater 2016 32 07 908 914 27087687 10.1016/j.dental.2016.03.013

[12] Dal Piva A MO Tribst J PM Venturini A B Survival probability of zirconia-reinforced lithium silicate ceramic: effect of surface condition and fatigue test load profile Dent Mater 2020 36 06 808 815 32360042 10.1016/j.dental.2020.03.029

[13] Sen N Isler S Microstructural, physical, and optical characterization of high-translucency zirconia ceramics J Prosthet Dent 2020 123 05 761 768 31383525 10.1016/j.prosdent.2019.05.004

[14] Tribst J PM Dal Piva A MO Borges A LS Anami L C Kleverlaan C J Bottino M A Survival probability, Weibull characteristics, stress distribution, and fractographic analysis of polymer-infiltrated ceramic network restorations cemented on a chairside titanium base: an in vitro and in silico study Materials (Basel) 2020 13 08 1879 10.3390/ma13081879 32316360 PMC7216243

[15] Ramos NdeC Campos T M Paz I S Microstructure characterization and SCG of newly engineered dental ceramics Dent Mater 2016 32 07 870 878 27094589 10.1016/j.dental.2016.03.018

[16] Brokos Y Stavridakis M Bortolotto Ibarra T Krejci I Evaluation of enamel thickness of upper anterior teeth in different age groups by dental cone beam computed tomography scan in vivo InterInt J Med Rev Case Rep. 2015 2 23 1396 1409

[17] Dal Piva A Contreras L Ribeiro F C Monolithic ceramics: effect of finishing techniques on surface properties, bacterial adhesion and cell viability Oper Dent 2018 43 03 315 325 29533718 10.2341/17-011-L

[18] Santos T DSA Abu Hasna A Abreu R T Fracture resistance and stress distribution of weakened teeth reinforced with a bundled glass fiber-reinforced resin post Clin Oral Investig 2021. Epub ahead of print 10.1007/s00784-021-04148-4 34435252

[19] Dal Piva A MO Tribst J PM Benalcázar Jalkh E B Anami L C Bonfante E A Bottino M A Minimal tooth preparation for posterior monolithic ceramic crowns: effect on the mechanical behavior, reliability and translucency Dent Mater 2021 37 03 e140 e150 33246664 10.1016/j.dental.2020.11.001

[20] Alves L MM Contreras L PC Campos T MB Bottino M A Valandro L F Melo R M In vitro wear of a zirconium-reinforced lithium silicate ceramic against different restorative materials J Mech Behav Biomed Mater 2019 100 103403 10.1016/j.jmbbm.2019.103403 31525551

[21] Anami L C Lima J M Valandro L F Kleverlaan C J Feilzer A J Bottino M A Fatigue resistance of Y-TZP/Porcelain crowns is not influenced by the conditioning of the intaglio surface Oper Dent 2016 41 01 E1 E12 26266655 10.2341/14-166-L

[22] Abu-Izze F O Ramos G F Borges A LS Anami L C Bottino M A Fatigue behavior of ultrafine tabletop ceramic restorations Dent Mater 2018 34 09 1401 1409 29934124 10.1016/j.dental.2018.06.017

[23] Fennis W MM Kuijs R H Kreulen C M Verdonschot N Creugers N H Fatigue resistance of teeth restored with cuspal-coverage composite restorations Int J Prosthodont 2004 17 03 313 317 15237878

[24] Sousa M P Tribst J PM de Oliveira Dal Piva A M Borges A LS de Oliveira S da Cruz P C Capacity to maintain placement torque at removal, single load-to-failure, and stress concentration of straight and angled abutments Int J Periodontics Restorative Dent 2019 39 02 213 218 30794257 10.11607/prd.3998

[25] Tribst J PM Dal Piva A MO Ausiello P De Benedictis A Bottino M A Borges A LS Biomechanical analysis of a custom-made mouthguard reinforced with different elastic modulus laminates during a simulated maxillofacial trauma Craniomaxillofac Trauma Reconstr 2021 14 03 254 260 34471482 10.1177/1943387520980237 PMC8385621

[26] Roscoe M G Noritomi P Y Novais V R Soares C J Influence of alveolar bone loss, post type, and ferrule presence on the biomechanical behavior of endodontically treated maxillary canines: strain measurement and stress distribution J Prosthet Dent 2013 110 02 116 126 23929373 10.1016/S0022-3913(13)60350-9

[27] Tribst J PM Dal Piva A MO Lo Giudice R The influence of custom-milled framework design for an implant-supported full-arch fixed dental prosthesis: 3D-FEA sudy Int J Environ Res Public Health 2020 17 11 4040 10.3390/ijerph17114040 32517097 PMC7313457

[28] Salas M MS Vargas-Ferreira F Ardenghi T M Peres K G Huysmans M D Demarco F F Prevalence and associated factors of tooth erosion in 8 -12-year-old Brazilian schoolchildren J Clin Pediatr Dent 2017 41 05 343 350 28872983 10.17796/1053-4628-41.5.343

[29] Vered Y Lussi A Zini A Gleitman J Sgan-Cohen H D Dental erosive wear assessment among adolescents and adults utilizing the basic erosive wear examination (BEWE) scoring system Clin Oral Investig 2014 18 08 1985 1990 10.1007/s00784-013-1175-0 24420504

[30] Vailati F Composite palatal veneers to restore a case of severe dental erosion, from minimally to non invasive dentistry: a 5-year follow-up case report It. J. Dent. Med. 2017 2 01 24 34

[31] Vailati F Vaglio G Belser U C Full-mouth minimally invasive adhesive rehabilitation to treat severe dental erosion: a case report J Adhes Dent 2012 14 01 83 92 21734973 10.3290/j.jad.a21852

[32] Muts E J van Pelt H Edelhoff D Krejci I Cune M Tooth wear: a systematic review of treatment options J Prosthet Dent 2014 112 04 752 759 24721500 10.1016/j.prosdent.2014.01.018

[33] Dal Piva A MO Tribst J PM Werner A Anami L C Bottino M A Kleverlaan C J Three-body wear effect on different CAD/CAM ceramics staining durability J Mech Behav Biomed Mater 2020 103 103579 10.1016/j.jmbbm.2019.103579 32090908

[34] Nishioka G Prochnow C Firmino A Fatigue strength of several dental ceramics indicated for CAD-CAM monolithic restorations Braz Oral Res 2018 32 e53 10.1590/1807-3107bor-2018.vol32.0053 29898029

[35] Tinschert J Schulze K A Natt G Latzke P Heussen N Spiekermann H Clinical behavior of zirconia-based fixed partial dentures made of DC-Zirkon: 3-year results Int J Prosthodont 2008 21 03 217 222 18548959

[36] Altan B Cinar S Tuncelli B Evaluation of shear bond strength of zirconia-based monolithic CAD-CAM materials to resin cement after different surface treatments Niger J Clin Pract 2019 22 11 1475 1482 31719267 10.4103/njcp.njcp_157_19

[37] Penteado M M Mendes Tribst J P Dal Piva A MO Archangelo K C Bottino M A Souto Borges A L Influence of different restorative material and cement on the stress distribution of ceramic veneer in upper central incisor Indian J Dent Res 2020 31 02 236 240 32436903 10.4103/ijdr.IJDR_150_18

[38] Gibbs C H Mahan P E Mauderli A Lundeen H C Walsh E K Limits of human bite strength J Prosthet Dent 1986 56 02 226 229 3463748 10.1016/0022-3913(86)90480-4

[39] Paphangkorakit J Osborn J W The effect of pressure on a maximum incisal bite force in man Arch Oral Biol 1997 42 01 11 17 9134111 10.1016/s0003-9969(96)00106-9

[40] Preis V Grumser K Schneider-Feyrer S Behr M Rosentritt M Cycle-dependent in vitro wear performance of dental ceramics after clinical surface treatments J Mech Behav Biomed Mater 2016 53 49 58 26313248 10.1016/j.jmbbm.2015.08.009

[41] Venturini A B Bohrer T C Fontana P E Fröhlich T T May L G Valandro L F Step-stress vs. staircase fatigue tests to evaluate the effect of intaglio adjustment on the fatigue behavior of simplified lithium disilicate glass-ceramic restorations J Mech Behav Biomed Mater 2021 113 104091 10.1016/j.jmbbm.2020.104091 33032009

[42] Gonzaga C C Cesar P F Miranda W G Jr Yoshimura H N Slow crack growth and reliability of dental ceramics Dent Mater 2011 27 04 394 406 21185074 10.1016/j.dental.2010.10.025

[43] Melo R M Pereira C Ramos N C Effect of pH variation on the subcritical crack growth parameters of glassy matrix ceramics Int J Appl Ceram Technol 2019 16 06 2449 2456

[44] Monteiro J B Riquieri H Prochnow C Fatigue failure load of two resin-bonded zirconia-reinforced lithium silicate glass-ceramics: effect of ceramic thickness Dent Mater 2018 34 06 891 900 29588077 10.1016/j.dental.2018.03.004

[45] Tribst J PM Dal Piva A MO Penteado M M Borges A LS Bottino M A Influence of ceramic material, thickness of restoration and cement layer on stress distribution of occlusal veneers Braz Oral Res 2018 32 e118 10.1590/1807-3107bor-2018.vol32.0118 30517427

[46] Meirelles L CF Pierre F Z Tribst J PM Pagani C Bresciani E Borges A LS Influence of preparation design, restorative material and load direction on the stress distribution of ceramic veneer in upper central incisorBraz Dent Sci 2021;24(03):

